# The Relationship of Objectively Measured Physical Activity and Sedentary Behaviour with Gestational Weight Gain and Birth Weight

**DOI:** 10.1155/2014/567379

**Published:** 2014-09-21

**Authors:** Anneloes E. Ruifrok, Ellen Althuizen, Nicolette Oostdam, Willem van Mechelen, Ben Willem Mol, Christianne J. M. de Groot, Mireille N. M. van Poppel

**Affiliations:** ^1^Department of Obstetrics, Gynaecology and Fertility, Faculty of Medicine, University of Amsterdam (AMC-UvA), Meibergdreef 9, 1105 AZ Amsterdam, The Netherlands; ^2^Department of Obstetrics and Gynaecology, Faculty of Medicine, VU University Medical Center, P.O. Box 7057, 1007 MB Amsterdam, The Netherlands; ^3^Department of Public and Occupational Health, EMGO^+^ Institute for Health and Care Research, VU University Medical Center, Van der Boechorststraat 7, 1081 BT Amsterdam, The Netherlands

## Abstract

*Objective*. To evaluate the relationship of physical activity (PA) and sedentary behaviour with gestational weight gain (GWG) and birth weight. *Design*. Combined data from two prospective studies: (1) nulliparous pregnant women without BMI restrictions and (2) overweight and obese pregnant women at risk for gestational diabetes. *Methods*. Daily PA and sedentary behaviour were measured with an accelerometer around 15 and at 32–35 weeks of gestation. The association between time spent in moderate-to-vigorous PA (MVPA) and in sedentary activities with GWG and birth weight was determined. Main outcome measures were GWG between 15 and 32 weeks of gestation, average GWG per week, and birth weight. *Results*. We studied 111 women. Early in pregnancy, 32% of women spent ≥30 minutes/day in at least moderate PA versus 12% in late pregnancy. No significant associations were found between time spent in MVPA or sedentary behaviour with GWG or birth weight. *Conclusions.* We found no relation between MVPA and sedentary behaviour with GWG or birth weight. The small percentage of women meeting the recommended levels of PA indicates the need to inform and support pregnant women to maintain regular PA, as there seems to be no adverse effect on birth weight and maintaining PA increases overall health.

## 1. Introduction

Excessive weight gain during pregnancy is associated with an increased risk of obstetrical, maternal, and fetal complications [1–4] and postpartum weight retention [5]. It increases the risk of obesity in children [5–7]. This contributes to the prevalence of women who are overweight or obese and increases the long-term risk of body weight-associated diseases, which impose a great pressure on health care [5, 8–12].

The American Institute of Medicine (IOM) updated their evidence-based guidelines for weight development during pregnancy in 2009 [13]. However, 53% of all women gain more weight than advised by the IOM. This is even more pronounced in women with overweight or obesity, with 68.9% and 59.8%, respectively, exceeding the recommendations [14].

Many trials have been conducted evaluating the effect of different lifestyle interventions on gestational weight gain (GWG) and adverse pregnancy outcomes, which were recently reviewed and combined in a meta-analysis [15, 16]. Combining results of 15 interventions consisting of physical activity (PA) alone did not result in a statistically significant effect on GWG and showed a very small but statistically significant reduction in mean birth weight.

However, it must be noted that the compliance with the interventions was either not assessed or insufficient in some trials. Furthermore, the total number of PA of participants was often not measured. Therefore, a possible compensation of PA levels outside of the intervention sessions could not be taken into account. In most studies that did measure total PA, this was done with questionnaires, which might often not give a valid estimate of PA levels [17]. All in all, although the design of intervention studies in general allows for conclusions with regard to causality, the mentioned methodological shortcomings hamper causal inference of PA leading to lower GWG and birth weight.

The relationship between sedentary behaviour and weight (gain) has been found in women and adolescent girls outside of pregnancy [18, 19]. In pregnancy, US women spent more than half of the monitored day in sedentary behaviour [20]. However, whether the amount of time spent sedentary influences weight gain or birth weight is currently unknown.

The primary aim of this study was therefore to examine the relationship of objectively measured physical activity with sedentary behaviour at two time points in pregnancy with gestational weight gain and birth weight in a population with a wide range of BMI.

## 2. Methods

We performed a secondary analysis of data of the randomized controlled trials performed by Althuizen et al. [21] (ISRCTN85313483) and Oostdam et al. [22, 23] (NTR1139). The interventions evaluated in the two trials were not effective in reducing gestational weight gain in the total study population [23, 24]. Data from both trials were combined and analysed as a cohort, as the study design and procedures were similar for both trials. All participants were healthy pregnant women, only the BMI's were different (no BMI restrictions (Althuizen) and overweight or obese (Oostdam)). In both trials, the participants were followed from 15 weeks of gestation until delivery, with objective measurements of physical activity and sedentary behaviour and body weight at baseline (around 15 weeks of gestation) and at 32–35 weeks of gestation. Birth weight was reported in questionnaires. The Medical Ethics Committee of the VU University Medical Center had approved design, protocols, and informed consent procedures of both studies.

The first cohort consisted of nulliparous pregnant women without BMI restrictions. A complete description of the inclusion and exclusion criteria has been published in Althuizen et al. [21]. The second cohort consisted of pregnant women with a BMI of >25 kg/m^2^ and at increased risk for GDM. Women were considered to be at an increased risk for GDM if they were obese (body mass index, BMI ≥ 30) or overweight (BMI ≥ 25) and had at least one of the three following characteristics: (1) history of macrosomia (offspring with a birth weight above the 97th percentile of gestational age), (2) history of GDM, or (3) first-grade relative with DM2. Exclusion criteria included recruitment after 20 weeks of gestation, age under 18 years, inadequate knowledge of the Dutch language, having been diagnosed with (gestational) diabetes mellitus before randomization, and severe chronic disease. A complete description of the inclusion and exclusion criteria has been published in Oostdam et al. [22]. For this paper, we excluded women with a twin pregnancy from the analyses.

The relationship of objectively measured physical activity (PA) and sedentary behaviour with gestational weight gain and birth weight was evaluated. The first measurements were at baseline (around 15 weeks of gestation), and the last measurements at 35 weeks of gestation in cohort 1 and at 32 weeks of gestation in cohort 2.

Maternal body weight was measured using calibrated electronic scales, with participants wearing only indoor clothing and no shoes. Prepregnancy weight was self-reported. On the first measurement, maternal body height was measured with bare feet and a (wall mounted) height scale. The measured height and weight were used to calculate BMI (kg/m^2^). For the purpose of this paper, gestational weight gain (GWG) was defined as the weight gained between the first and the last measurements (kg). The neonatal outcome was birth weight, reported by the women in a questionnaire six weeks postpartum.

Daily physical activity (PA) was measured objectively using an accelerometer (ActiTrainer accelerometer; ActiGraph, Pensacola, FL, USA). This accelerometer is a compact, lightweight, and uniaxial device that measures and records time-varying acceleration. Days with at least 8-hour registration time were used. Total counts per minute were converted into light, moderate, and vigorous PA (100 to 2019 counts/min for light PA, 2020 to 5998 counts/min for moderate PA and ≥5999 counts/min for vigorous PA) [25]. Sedentary behaviour was defined as <100 counts/min. In subsequent analysis, time spent by the participants was measured as a percentage of total registration time. These subsequent analysis were time spent in any physical activity (total PA), in moderate-to-vigorous physical activity (MVPA) and sedentary time.

Ethnicity was derived from the country of birth of the participant's parents. An individual was considered to be white European if both parents were born in Europe (with the exception of Turkey and Morocco; two groups with a higher risk for GDM) or North America. Furthermore, level of education was assessed as the highest level an individual reported to have achieved, which was then divided into lower, middle, or higher educational levels. Moreover, participants were asked to report on their status of employment (yes or no). Gestational age at delivery was self-reported.

The maternal characteristics of the study are presented as means and standard deviations for continuous variables and as percentages for ordinal variables. For the outcomes gestational weight gain and birth weight, standard linear regression analysis was used to test the association between the percentage of time spent sedentary or in physical activity at baseline and between the change in MVPA and sedentary time from 15 weeks to 32–35 weeks of gestation and the outcome. Regression models were controlled for allocation to intervention or control group, the difference in gestational age between the two measurements (weight gain and weight gain/week) or gestational age at birth (birth weight), BMI at first measurement during pregnancy, and parity and age.

The analyses were checked for effect modification by age and BMI. It was concluded that effect modification was present in case the *P* value of the interaction term was significant (*P* < 0.10). All analyses were performed using SPSS 20.0 (Statistical Package for the Social Sciences, SPSS Inc., Chicago, IL, USA) for windows, and the level of significance was set to <0.05.

## 3. Results

A total of 390 women were included in the two trials: 269 in cohort 1 and 121 women in cohort 2. Of these women, 139 completed both baseline and late pregnancy data collection. Due to lack of compliance of the participants, data on objectively measured PA and sedentary behaviour were available for 111 (80%) women with a singleton pregnancy. They comprised the study sample for the analyses. The baseline characteristics of the study population and the outcome measures are presented in Table 1. The mean GWG was 10.3 (SD 4.3) kg, with an average of 0.55 (SD 0.22) kg per week and mean birth weight was 3545 (SD 441) g.

Total daily physical activity (PA) measured with accelerometers at baseline showed an average of 286 (SD 103) minutes per day (range 45 to 512 minutes per day). At 32–35 weeks of gestation the mean total PA was 273 (SD 103) minutes/day. At both times this accounted for 35% of registration time. Overall, the minutes spent per day performing moderate and vigorous PA (MVPA) reduced during the pregnancy. At baseline, the mean number of minutes of moderate and vigorous PA spent per week was 24 (SD 16) minutes/day. At 32–35 weeks of gestation the mean number of minutes of moderate and vigorous PA performed per week had decreased to 18 (SD 22) minutes/day. This was a drop from 3% to 2% of the total registration time. At baseline, 31% of the women spent ≥ 30 minutes/day in MVPA and therefore met the guidelines of the ACOG for sufficient PA [26]. At 32–35 weeks, this proportion dropped to 12% of the women.

Sedentary behaviour remained relatively stable during pregnancy, with women spending more than 500 minutes/day (65% of the registration time) sedentary at both time points.

No statistically significant association was found between MVPA or sedentary behaviour at 15 weeks with GWG or GWG/week (Table 2). Also no significant associations were found for changes in PA and sedentary behaviour from 15 to 32–35 weeks of gestation. With birth weight as outcome, also no significant associations were found with the percentage of time in MVPA or sedentary behaviour (Table 2). Gestational age was not related to any PA or sedentary behaviour parameter (data not shown). No effect modifications of age or BMI were found.

## 4. Discussion

In this study, the association between objectively measured moderate to vigorous PA and/or sedentary behaviour with gestational weight gain (GWG) and birth weight was examined. We found that neither PA nor sedentary behaviour had an association with GWG or birth weight.

This is in line with the findings of a meta-analysis of 15 trials, showing no significant reduction in GWG in trials evaluating PA interventions [15, 16]. In the same meta-analysis, the pooled result of 14 PA trials showed a small (−60 g) but significant reduction in birth weight [15, 16]. A different meta-analysis, by Streuling et al. [27], showed a reduction of 61 g (CI −1.17 to −1.06) in GWG in the group receiving a PA intervention. Our sample size was very likely insufficient to detect such a small reduction in birth weight. It was certainly insufficient to study the effect of PA on the number of babies born small or large for gestational age.

The relationship between objectively measured sedentary behaviour and GWG or birth weight has not been studied so far, to our knowledge. Although outside pregnancy, sedentary behaviour is related to weight status in girls and women [18, 19], we could not establish an association with GWG or birth weight. This would indicate that trying to reduce sedentary behaviour in pregnant women would not likely lead to reduced GWG or changes in birth weight.

The data used for this study were collected in two separate trials [21, 22], and the results presented here are from secondary analysis of the data. However, since the interventions neither had an effect on GWG nor on birth weight, the design and procedures were similar for both trials, and all participants were healthy pregnant women [21, 23], we felt justified in analysing the data as a cohort. By combining the two datasets, there was a wider variation in PA levels in the data, which is needed for assessing an association with outcomes. However, the participants did not include women who participated in regular vigorous exercise. Our results can thus not be extrapolated to women who continue to participate in competitive exercise or elite sports during pregnancy.

The data on birth weight were self-reported by the mothers about six weeks after birth. This might have led to some inaccuracy in our outcome measure. Other studies showed, however, that self-reports of birth weight are accurate [28, 29], with small, clinically nonrelevant differences between birth weight in medical records and self-reports [29].

The data on physical activity and sedentary behaviour were objectively measured, reducing the reporting bias and increasing the accuracy of the results. The accelerometer, used for monitoring the amount of PA and sedentary behaviour, is a uniaxial device that measures and records vertical acceleration. In the Netherlands, many women cycle and continue to do so during pregnancy. The accelerometer does not record such activity well due to its uniaxial nature. Therefore the amount of PA measured might be underestimated. Furthermore, it has been shown that accelerometers might be less valid in pregnancy, mostly because of slower walking speeds of the women [30]. However, objective measurement of PA is to be preferred over using self-reported PA since most questionnaires show poor validity in pregnancy [31]. In this study, nutritional intake was not taken into account, which might have confounded the results presented in this paper. Weight gain is a function of energy expenditure through physical activity and metabolism, as well as energy intake from food and drink consumption. Physical activity levels and sedentary time reflect only one half of the equation. Physical activity may affect appetite and food intake during pregnancy; furthermore, women who are health oriented may be more physically active and eat more healthily. Future studies are needed in which both sides of the energy balance are taken into account in relation to GWG and birth weight. And although we did not find an interaction with BMI, it might be useful to study the relationship between PA and sedentary behaviour in different BMI categories separately.

## 5. Conclusion

This study showed that PA is not associated with GWG or birth weight, and also sedentary behaviour did not seem to contribute to GWG or birth weight of the infant. The findings regarding sedentary behaviour are new and need to be confirmed with studies using a design better suitable for studying causal relationships, such as randomized trials. The findings with regard to PA are not in line with the results of recent meta-analyses, and also here more research is needed to assess the relationship of objectively measured PA with GWG and birth weight. Another important finding is that only a small proportion of our pregnant women met the ACOG guidelines for sufficient MVPA [26] in early pregnancy (31%) and even fewer at the end of pregnancy (13%). This is much lower than the 58% of women 20–40 years of age meeting similar guidelines in the general Dutch population in 2010 according to the Dutch Bureau of Statistics (http://statline.cbs.nl). This indicates that pregnant women need to be better informed and advised about maintaining PA levels throughout pregnancy.

In summary, we have conducted analysis estimating the relationship between PA and sedentary behaviour and GWG and birth weight. Key finding of this study was that neither behaviour, physical or sedentary, at any time during pregnancy was associated with gestational weight gain or birth weight of the newborn.

## 6. Practical Implications

- Physical activity or sedentary behaviour does not seem to contribute to GWG.
- Physical activity or sedentary behaviour does not seem to affect the birth weight of the newborn.
- A small proportion of pregnant women meet the ACOG guidelines for sufficient MVPA in pregnancy, and pregnant women need to be informed and advised about physical activity throughout pregnancy.

## Figures and Tables

**Table 1:** Characteristics of the study sample.

	Total population (*n* = 111)	
Age, years, mean (SD)	29.6	(3.8)
Ethnicity, *N* (%)		
White European	95	86%
Non-White	16	14%
Nulliparous, *N* (%)	81	73%
BMI at 15 wks (kg/m^2^), mean (SD)	27.0	(5.5)
BMI category at 15 weeks, *N* (%)		
Normal weight	54	49%
Overweight	25	22%
Obese	32	29%
Gestational age at birth, weeks, mean (SD)	40.2	(1.2)
Gestational weight gain, kg, mean (SD)	10.3	(4.2)
Gestational weight gain per week, kg, mean (SD)	0.55	(0.22)
Birth weight, g, mean (SD)	3541	(429)
Total PA at 15 wks, mins/day, mean (SD)	286	(103)
% of registration time		35%
Total PA at 32–35 wks, mins/day, mean (SD)	273	(103)
% of registration time		35%
MVPA at 15 wks, min/day, mean (SD)	24	(16)
% of registration time		3%
MVPA at 32–35 wks, min/day, mean (SD)	18	(22)
% of registration time		2%
Sedentary behaviour at 15 wks, min/day, mean (SD)	530	(170)
% of registration time		65%
Sedentary behaviour at 32–35 wks, min/day, mean (SD)	505	(173)
% of registration time		65%

MVPA: moderate to vigorous physical activity; PA: physical activity; SD: standard deviation.

**Table 2:** Associations between PA, sedentary behaviour, gestational weight gain, and birth weight.

	Gestational weight gain between 15 and 32–35 weeks		Gestational weight gain per week		Birth weight	
	Beta	95% CI	Beta	95% CI	Beta	95% CI
15 weeks of gestation∗						
% MVPA	−0.07	−0.48; 0.34	−0.002	−0.02; 0.02	26.93	−14.79; 68.65
% Sedentary behaviour	−0.07	−0.15; 0.01	−0.004	−0.01; 0.001	2.45	−5.53; 10.42
Change from 15 to 32–35 weeks of gestation∗						
% MVPA	−0.16	−0.47; 0.15	−0.01	−0.03; 0.01	8.10	−23.83; 68.44
% Sedentary behaviour	−0.02	−0.12; 0.07	−0.001	−0.01; 0.004	0.59	−8.91; 10.09

*Moderate to vigorous PA (MVPA) and sedentary behaviour were entered into the same model, controlled for intervention group, age, parity, BMI, and (change in) gestational age. The model with MVPA and sedentary behaviour at 32–35 weeks was also controlled for baseline values and therefore reflects the betas for the changes in MVPA and sedentary behaviour.

CI: confidence interval; MVPA: moderate to vigorous physical activity; PA: physical activity.
